# Fault Detection in Real-Time Kinematic Positioning Using Multiple Reference Stations

**DOI:** 10.3390/s25154653

**Published:** 2025-07-27

**Authors:** Euiho Kim, Soomin Lee

**Affiliations:** 1 Department of Mechanical & System Design Engineering, Hongik University, Seoul 04066, Republic of Korea; 2 Department of Mechanical Engineering, Hongik University, Seoul 04066, Republic of Korea; c3111605@hongik.ac.kr

**Keywords:** RTK, fault monitoring, GNSS, innovation

## Abstract

Multiple-reference-station-based real-time kinematics (MR-RTK) is an advanced RTK technique that leverages global navigation satellite system (GNSS) measurements from multiple reference stations and their known baselines. This study investigates the fault detection capabilities of MR-RTK by employing additional measurements from continuously operating reference stations (CORSs) to evaluate the probability of missed detection. The proposed method was validated using test data from a ground rover and a few CORSs within a 10 km radius. The test results show that the missed detection probability decreased by up to 55.0% as the number of reference stations increased up to four.

## 1. Introduction

Global navigation satellite system (GNSS) real-time kinematics (RTK) achieves centimeter-level positioning accuracy by leveraging double-difference (DD) measurements of carrier phase observations [1,2]. The accurate resolution of ambiguities in GNSS carrier phase measurements is critical to RTK performance and is typically accomplished using methods such as the integer least squares (ILS) or the least squares ambiguity decorrelation adjustment (LAMBDA) algorithm [3,4]. Several studies have proposed modifications to the LAMBDA method to enhance speed and reliability in ambiguity resolution [5]. For instance, the authors in [6] incorporated quadratic equality constraints into the ILS problem by employing multiple receivers. Similarly, Teunissen and Giorgi utilized baseline constraints between receivers to improve the LAMBDA method for GNSS attitude determination [7,8]. Additional approaches include applying multivariate constraints based on known conditions [9] and developing a general multivariate formulation for multi-antenna GNSS attitude determination [10]. Moreover, Giorgi et al. demonstrated that the multivariate constrained LAMBDA method can significantly enhance positioning performance, highlighting its potential for high-precision applications [11]. Wang et al. introduced a novel algorithm for the partial resolution of ambiguity among reference stations utilizing a robust extended Kalman filter combined with the LAMBDA method and a joint test involving the ratio test and bootstrapping success rate index solver [12]. Kim et al. proposed the use of an ionosphere-free combination and the application of a Kriging-based weighting model to mitigate tropospheric delay, thereby improving integer ambiguity resolution rates in long-baseline RTK networks [13]. Yuan et al. developed a resilient ambiguity resolution strategy based on a batch best integer equivariant (BIE) estimator, which integrates integer rounding, bootstrapping, integer least squares (ILS), and BIE to enhance ambiguity resolution in urban environments [14].

Expanding on the concept of the known distances between two receivers, recent developments have introduced the use of multiple reference receivers—an approach known as multiple-reference RTK (MR-RTK) positioning. In MR-RTK, the accurately known antenna coordinates of multiple reference receivers are used to compute DD integer ambiguities. This extra information effectively increases the number of available measurements, which is especially beneficial in environments with limited satellite visibility [15,16,17,18,19]. Specifically, in [15,16], the authors incorporated known baselines and integer ambiguities among receivers as linear constraints in ambiguity resolution. Similarly, Wu et al. investigated the robustness of MR-RTK for both float and fixed integer solutions in the presence of measurement biases [17], while He et al. demonstrated the kinematic positioning performance of MR-RTK on a moving ship platform equipped with multiple GNSS antennas [18]. In addition, Kim et al. introduced a Kalman filter-based MR-RTK method for the precise positioning of multiple unmanned aerial vehicles [19]. Liu et al. presented positioning error bounds for MR-RTK based on the Cramér–Rao Bound (CRB) and proposed strategies for optimizing the geometry of reference stations [20].

Recent research on multi-antenna-aided positioning has targeted applications such as autonomous driving, infrastructure monitoring, and urban navigation. In [21,22], researchers integrated multiple GNSS antennas with a consumer-grade inertial measurement unit to enhance navigation performance in urban areas. Furthermore, Yuan et al. developed a multi-antenna setup that achieved millimeter-level positioning accuracy for railway track monitoring [23]. In [24], scenarios in which a priori integer ambiguities were unavailable were considered, and the geometric relationships among the antennas were utilized to resolve ambiguities, thereby providing lane-level accuracy for vehicles under challenging conditions.

**Figure 1:**
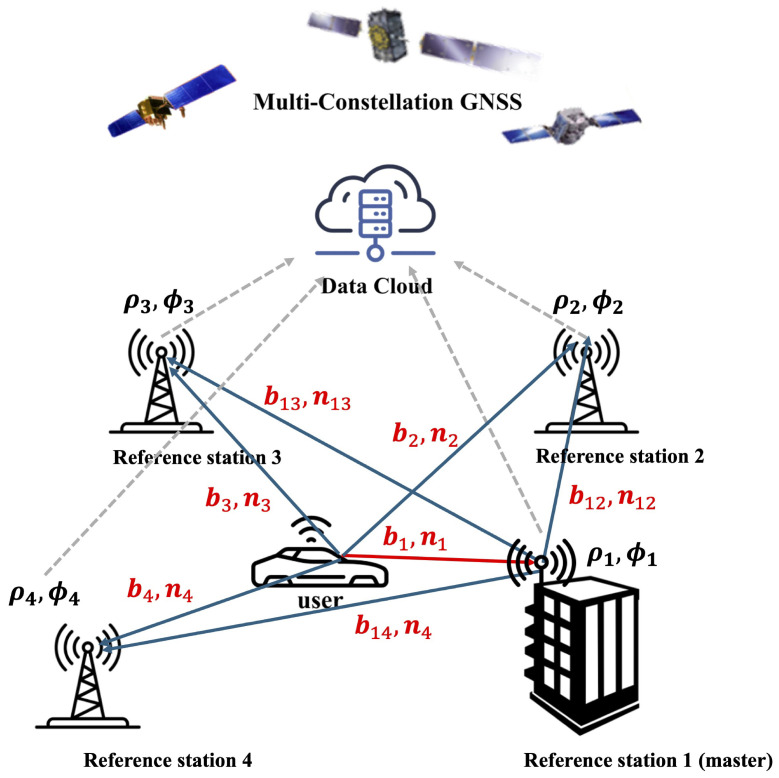
Conceptual image of MR-RTK with multiple reference stations. $b_{i}$ and $n_{i}$ are the baselines and DD integer ambiguities, respectively, from the user to reference station *i*. $b_{ij}$ and $n_{ij}$ are the baselines and DD integer ambiguities, respectively, associated with reference stations *i* and *j*. $\rho_{i}\;\mathrm{and}\;\phi_{i}$ are the code and carrier phase measurements at reference station *i*.

While previous studies on MR-RTK focused on improving the integer ambiguity resolution process, this study investigated the fault detection capability of MR-RTK by leveraging publicly available reference stations, i.e., continuously operating reference stations (CORSs), situated several kilometers from the user receiver. In the proposed MR-RTK setup, raw GNSS measurements, a priori integer ambiguities, and the precise coordinates of the reference stations are uploaded to a data cloud accessible by MR-RTK users. Figure 1 illustrates the concept of MR-RTK, in which a master station is used to estimate the relative baseline with the MR-RTK user. This architecture not only allows for additional measurements but also enables measurement fault monitoring because the CORSs are usually located in GNSS-benign environments free from excessive multipath or non-line-of-sight signals. This characteristic is particularly advantageous for users operating in GNSS-challenging environments where RTK performance is otherwise degraded [25].

This study presents a fault detection monitor based on the MR-RTK architecture, utilizing the innovations produced by the MR-RTK Kalman filter. The innovation of a Kalman filter refers to the discrepancy between the predicted sensor measurements—based on the system model—and the actual sensor observations. The chi-squared statistic derived from whitened innovations is commonly employed as the test metric in statistical hypothesis testing. To highlight the fault detection capabilities of the proposed MR-RTK monitor, this study analyzes how the chi-squared distributions under nominal and faulty conditions vary with the number of reference stations, using both theoretical analysis and experimental validation. Furthermore, the monitoring performance is quantitatively assessed by evaluating the missed detection probability in scenarios where faults are deliberately injected into the raw GNSS measurements.

The remainder of this paper is organized as follows: Section 2 describes the measurement model and the augmentation process in MR-RTK. In Section 3, we detail the extended Kalman filter (EKF) process for MR-RTK, introduce an innovation-based fault detection monitor, and evaluate its performance in terms of the missed detection probability as a function of the number of reference stations. Section 4 presents the test results obtained using ground rover data to demonstrate the proposed MR-RTK method, including a performance assessment performed by injecting measurement faults in satellites. Then, conclusions are provided. The symbols and abbreviations used in the paper are listed in Table 1.

## 2. Measurement Model for MR-RTK

RTK utilizes DD measurements to eliminate common errors—such as satellite clock bias, receiver clock bias, and other challenging-to-estimate biases, such as atmospheric delays—when the user and the reference receiver are in close proximity. However, for longer baselines, additional atmospheric corrections (e.g., differential correction data) must be applied. The DD code and carrier phase measurements between a user *u* and reference stations 1–3 in short baselines can be modeled as follows:

(1) $$\begin{array}{cc} E(\phi_{1}) & =Gb_{1}+\Lambda n_{1},E\left(\rho_{1}\right)=Gb_{1}\\ E(\phi_{2}) & =Gb_{2}+\Lambda n_{2},E\left(\rho_{2}\right)=Gb_{2}\\ E(\phi_{3}) & =Gb_{3}+\Lambda n_{3},E\left(\rho_{3}\right)=Gb_{3} \end{array}$$

where $\phi_{i}={\left[\begin{array}{ccccccc} \phi_{i,1}^{1,2} & \ldots & \phi_{i,1}^{1,m+1} & \ldots & \phi_{i,f}^{1,2} & \ldots & \phi_{i,f}^{1,m+1} \end{array}\right]}^{T}$ is the DD carrier phase measurement vector and $\rho_{i}={\left[\begin{array}{ccccccc} \rho_{i,1}^{1,2} & \ldots & \rho_{i,1}^{1,m+1} & \ldots & \rho_{i,f}^{1,2} & \ldots & \rho_{i,f}^{1,m+1} \end{array}\right]}^{T}$ is the DD code measurement; E(·) is the expectation operator; *i* is a reference receiver index and *f* indicates the GNSS carrier frequency; the superscript indicates a pair of m+1 satellite indices having 1 as a pivot satellite, $b_{i}={\left[\begin{array}{ccc} b_{i,x} & b_{i,y} & b_{i,z} \end{array}\right]}^{T}$ is the baseline vector between a user and reference stations, and $n_{i}={\left[\begin{array}{ccccccc} n_{i,1}^{1,2} & \ldots & n_{i,1}^{1,m+1} & \ldots & n_{i,f}^{1,2} & \ldots & n_{i,f}^{1,m+1} \end{array}\right]}^{T}$ is the corresponding DD integer ambiguity vector; $\Lambda =\lambda ⊗I_{m}$ is a wavelength matrix with $\lambda =diag\left(\begin{array}{cccc} \lambda_{1}, & \lambda_{2}, & \ldots , & \lambda_{f} \end{array}\right)$ and ⊗ denotes the Kronecker product; $I_{m}$ is an identity matrix with a dimension of m×m; $G=e_{f}⊗g$ is a DD user-to-satellite line-of-sight matrix for all frequencies; $e_{f}$ is a column vector of 1s corresponding to the number of *f* used in the measurements; and g is a DD user-to-satellite line-of-sight matrix for an *f*.

The relationships of the baseline and integer ambiguity vectors between the reference receivers and the user in a short baseline can be written as

(2) $$\begin{array}{c} b_{2}=b_{1}+b_{21}\\ b_{3}=b_{1}+b_{31}\\ n_{2}=n_{1}+n_{21}\\ n_{3}=n_{1}+n_{31} \end{array}$$

where $b_{ij}$ is the baseline vector between reference stations *i* and *j*, and $n_{ij}$ denotes the DD integer ambiguities of the carrier phases measurements in reference stations *i* and *j*. By using the a priori information of $b_{21}$, $b_{31}$, $n_{21}$, and $n_{31}$, Equation (1) can be transformed as

(3) $$\begin{array}{cc} E(\phi_{1}) & =Gb_{1}+\Lambda n_{1}\\ E(\phi_{2}) & =G\left(b_{21}+b_{1}\right)+\Lambda \left(n_{21}+n_{1}\right)\\ E(\phi_{3}) & =G\left(b_{31}+b_{1}\right)+\Lambda \left(n_{31}+n_{1}\right)\\ E(\rho_{1}) & =Gb_{1}\\ E(\rho_{2}) & =G(b_{21}+b_{1})\\ E(\rho_{3}) & =G(b_{31}+b_{1}) \end{array}$$

(4) $$\begin{array}{cc} E(\left[\begin{array}{ccc} \phi_{1} & \phi_{2} & \phi_{3} \end{array}\right]) & =G\left[\begin{array}{ccc} 0 & b_{21} & b_{31} \end{array}\right]+G\left[\begin{array}{ccc} b_{1} & b_{1} & b_{1} \end{array}\right]\\ \; & +\Lambda \left[\begin{array}{ccc} 0 & n_{21} & n_{31} \end{array}\right]+\Lambda \left[\begin{array}{ccc} n_{1} & n_{1} & n_{1} \end{array}\right]\\ E(\left[\begin{array}{ccc} \rho_{1} & \rho_{2} & \rho_{3} \end{array}\right]) & =G\left[\begin{array}{ccc} 0 & b_{21} & b_{31} \end{array}\right]+G\left[\begin{array}{ccc} b_{1} & b_{1} & b_{1} \end{array}\right] \end{array}$$

By subtracting the a priori baselines and DD integer ambiguities, Equation (4) can be written as

(5) $$\begin{array}{cc} E(\Phi -GB_{1}-\Lambda N_{1}) & =G\left[\begin{array}{ccc} b_{1} & b_{1} & b_{1} \end{array}\right]+\Lambda \left[\begin{array}{ccc} n_{1} & n_{1} & n_{1} \end{array}\right]\\ E(\mathrm{Pr}-GB_{1}) & =G\left[\begin{array}{ccc} b_{1} & b_{1} & b_{1} \end{array}\right] \end{array}$$

where $\Phi =\left[\begin{array}{ccc} \phi_{1} & \phi_{2} & \phi_{3} \end{array}\right]$ and $\mathrm{Pr}=\left[\begin{array}{ccc} \rho_{1} & \rho_{2} & \rho_{3} \end{array}\right]$ are matrices consisting of DD carrier and code phase measurements, respectively; $B_{1}=\left[\begin{array}{ccc} 0 & b_{21} & b_{31} \end{array}\right]$ is the matrix with known baseline vectors between reference receivers; and $N_{1}=\left[\begin{array}{ccc} 0 & n_{21} & n_{31} \end{array}\right]$ is the matrix with the a priori DD integer ambiguity vector of the baselines.

Equation (5) can be written in vector form as follows:

(6) $$\begin{array}{cc} E(y_{\Phi }) & =\bar{\Lambda }n_{1}+\bar{G}b_{1}\\ E(y_{\mathrm{Pr}}) & =\bar{G}b_{1} \end{array}$$

where $y_{\Phi }=vec\left(\begin{array}{c} \Phi -GB_{1}-\Lambda N_{1} \end{array}\right)$ and $y_{\mathrm{Pr}}=vec\left(\begin{array}{c} \mathrm{Pr}-GB_{1} \end{array}\right)$, with vec being a vectorizing operator; $\bar{\Lambda }=e_{r}⊗\Lambda$ and $\bar{G}=e_{r}⊗G$, with ⊗ being the Kronecker product; and $e_{r}$ is a column vector of 1s whose number of rows is the same as the number of reference receivers.

## 3. EKF and Fault Monitoring in MR-RTK

For the estimation of baseline and integer ambiguities, a state vector is defined as follows:

(7) $$\begin{array}{cc} x_{k} & ={\left[\begin{array}{ccc} b_{1,k}^{T} & v_{1,k}^{T} & n_{1,k}^{T} \end{array}\right]}^{T} \end{array}$$

where $v_{1}$ is the velocity of $b_{1}$. The discrete extended Kalman filter (EKF) for state estimation is expressed as

(8) $$\begin{array}{cc} {\hat{x}}_{k}^{-} & =F_{k-1}^{k}{\hat{x}}_{k-1}^{+}\\ P_{k}^{-} & =F_{k-1}^{k}P_{k}^{+}{F_{k-1}^{k}}^{T}+Q_{k-1}^{k} \end{array}$$

where ${\hat{x}}_{k}$ and $P_{k}$ are the estimated states and its variance–covariance (VC) matrix at epoch *k*, respectively; the superscripts − and + denote prediction and measurement updates, respectively; $Q_{k-1}^{k}$ is the VC matrix of the system noise; and $F_{k-1}^{k}$ is the state transition matrix from epoch k−1 to *k*.

From Equation (6), EKF measurement vector $y_{k}$ is

(9) $$\begin{array}{cc} y_{k} & =\left[\begin{array}{c} y_{\Phi ,k}\\ y_{\mathrm{Pr},k} \end{array}\right] \end{array}$$

which is a nonlinear function of $x_{k}$ such that $z_{k}=h\left(x_{k}\right)$. The measurement matrix, $H_{k}$, is a Jacobian matrix of $z_{k}$ at ${\hat{x}}_{k}^{-}$ and can be written as

(10) $$\begin{array}{cc} H_{k} & =\left[\begin{array}{c} e_{r}⊗H_{\Phi ,k}\\ e_{r}⊗H_{Pr,k} \end{array}\right]\\ H_{\Phi ,k} & =\left[\begin{array}{cc} G_{k} & \Lambda_{k} \end{array}\right]\\ H_{Pr,k} & =\left[\begin{array}{cc} G_{k} & 0 \end{array}\right] \end{array}$$

where $H_{\Phi }$ and $H_{Pr}$ are the measurement matrices for the DD carrier phase measurements and DD code measurements, respectively. With the above formulations and measurement of the VC matrix of $R_{k}$, a typical EKF can be implemented to estimate the float solution of $n_{1,k}$. Moreover, LAMBDA algorithms can also be used to find the corresponding fixed integers. For a more detailed description of an EKF-based MR-RTK system, readers can refer to [19].

Fault detection and exclusion are often implemented for the sanity check of position solutions. In EKF implementation, an innovation- or residual-based fault monitor is preferred [26,27]. In this section, we discuss an innovation-based fault detection technique for MR-RTK. In this scheme, reference stations are assumed to be located in a GNSS-benign environment such that their measurements are fault-free, while measurement faults may occur on the user side.

Assuming that there are *m* receivers, the DD measurements in Equation (9) at epoch *k* can be expressed as $y_{k}={\left[\begin{array}{c} y_{k_{\phi 1}}^{T},\ldots ,y_{k_{\phi m}}^{T},y_{k_{Pr_{1}}}^{T},\ldots ,y_{k_{Pr_{m}}}^{T} \end{array}\right]}^{T}$. The EKF innovation vector is computed as follows:

(11) $$\begin{array}{cc} z_{k} & =y_{k}-h_{k}\left(x_{k}^{-}\right). \end{array}$$

The corresponding innovation covariance matrix is

(12) $$\begin{array}{cc} S_{k} & =H_{k}P_{k}^{-}H_{k}^{T}+R_{k} \end{array}$$

where $H_{k},P_{k}$, and $R_{k}$ are the user-to-satellite geometry matrix, the state covariance matrix, and the measurement covariance matrix, respectively. Because $z_{k}$ can be highly correlated, it should be whitened, i.e., decorrelated and normalized, for proper fault detection. The whitened innovation test statistics, $q_{k}$, is computed as follows:

(13) $$\begin{array}{cc} q_{k} & =z_{k}^{T}S_{k}^{-1}z_{k}. \end{array}$$

In nominal cases, $q_{k}$ can be assumed to follow a chi-squared distribution such that $q_{k}∼\chi^{2}\left(m\cdot \nu \right)$, where ν is the degree of freedom associated with the DD measurements of the user and one reference station and *m* is the number of reference receivers. For simplicity, we assume that all reference stations receive measurements from the same set of satellites and frequencies.

When there is one reference station (master) and a user has a measurement fault from a single satellite, the innovation vector can be expressed as

(14) $$\begin{array}{ccc} z_{k,f} & = & z_{k,n}^{1}+z_{k,f}^{1}\\ & = & z_{k,n}^{1}+{\left[\begin{array}{c} 0,\ldots ,f_{z}^{1,i},\ldots ,0 \end{array}\right]}^{T} \end{array}$$

where $z_{k,n}^{1}$ is an innovation vector in nominal cases and $f_{z}^{1,i}$ is an innovation element that occurred owing to the measurement faults in the $i^{th}$ satellite. The superscript 1 indicates innovation derived from the DD measurements from the user and master reference station. In this case, the corresponding test statistics, $q_{k}$, follows a noncentral chi-squared distribution such that $q_{k}∼\chi^{2}\left(\nu ,\tau_{1}\right)$, where $\tau_{1}$ is the noncentral parameter contributed by the measurement fault. For MR-RTK with two reference stations (master and reference station 2), the innovation vector in Equation (14) changes to

(15) $$\begin{array}{ccc} z_{k,f} & = & \left[\begin{array}{c} z_{k,n}^{1}+z_{k,f}^{1}\\ z_{k,n}^{2}+z_{k,f}^{2} \end{array}\right]\\ & = & \left[\begin{array}{c} z_{k,n}^{1}+{\left[\begin{array}{c} 0,\ldots ,f_{z}^{1,i},\ldots ,0 \end{array}\right]}^{T}\\ z_{k,n}^{2}+{\left[\begin{array}{c} 0,\ldots ,f_{z}^{2,i},\ldots ,0 \end{array}\right]}^{T} \end{array}\right]. \end{array}$$

**Figure 2:**
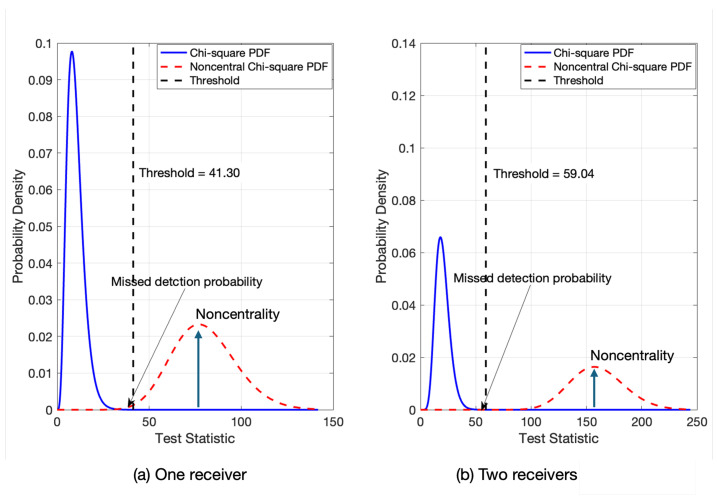
Illustration of probability density functions of the central and noncentral chi-squared distributions with (**a**) one and (**b**) two receivers in MR-RTK.

The corresponding test statistics, $q_{k}$, follows a noncentral chi-squared distribution such that $q_{k}∼\chi^{2}\left(2\nu ,\tau_{1}+\tau_{2}\right)$, where $\tau_{2}$ is the noncentrality parameter contributed by the second reference station. Figure 2 illustrates the benefit derived from the fault detection capability of the MR-RTK system. In this figure, ν is set to 10, and $\tau_{1}\;\mathrm{and}\tau_{2}$ are set to 70; the threshold was derived from a false alert rate of $10^{-5}$. Figure 2a,b depict the central and noncentral chi-squared probability density functions (PDFs) of $q_{k}$ for MR-RTK with one and two receivers, respectively. Owing to the larger distance from the threshold and the noncentrality parameters in MR-RTK with two receivers, the missed detection ratio is significantly reduced. The reliable fault detection capability of the MR-RTK system was verified with test measurements, as detailed in the next section.

## 4. Test Results of Proposed MR-RTK Fault Detection Method

**Figure 3:**
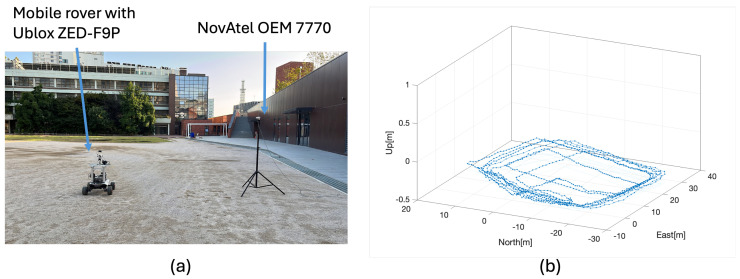
(**a**) Mobile rover and master station with NovAtel OEM7700 GNSS receiver. (**b**) Mobile rover trajectory during the tests.

**Figure 4:**
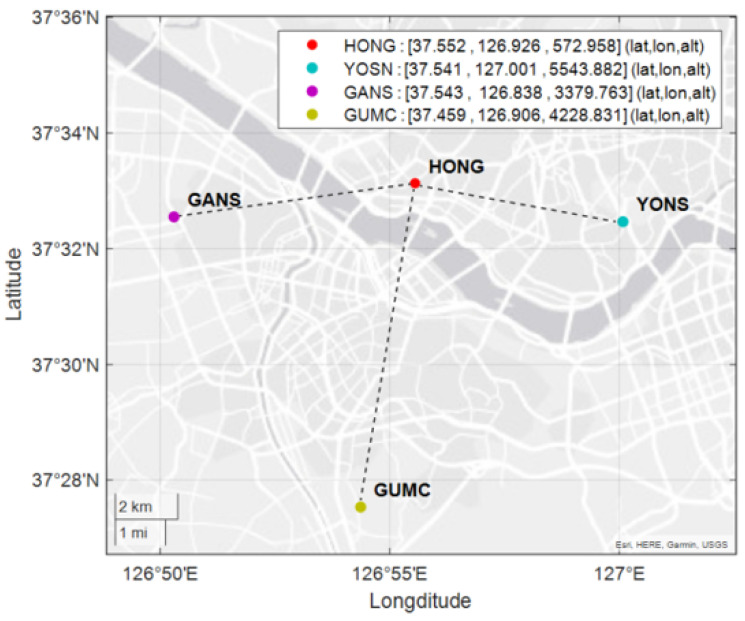
Locations and coordinates of CORSs. HONG is used as the master reference station.

To evaluate the fault detection capability of the MR-RTK system, experiments were conducted on 5 October 2023, in an urban environment using a rover equipped with a ZED−F9P receiver, Ublox, Thalwil, Switzerland. Figure 3 illustrates the experimental configuration, which includes a mobile rover and a NovAtel OEM7700 receiver, Hexagon, Calgary, AB, Canada, operating as the master reference station, as well as the rover’s trajectory over a 15-minute maneuvering period during the test. The MR-RTK experiment was performed using raw GNSS measurements collected from the rover and three reference stations, all located within a 10 km baseline. Details of the reference stations are summarized in Table 2, and their geographical distribution is shown in Figure 4. The GNSS receivers were configured to track signals from both the GPS and Galileo constellations. A total of 10 satellites, commonly visible to all reference stations and the rover, were utilized in the analysis.

**Figure 5:**
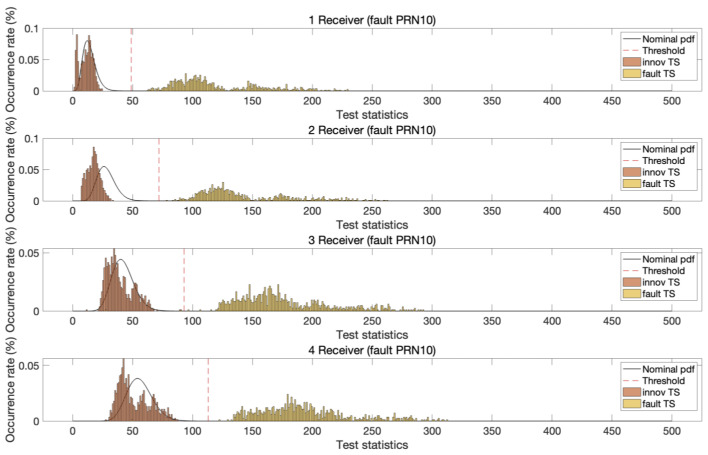
The chi-squared innovation test statistics (m^2^) of MR-RTK for PRN 10 GPS satellite under both nominal and fault conditions with a detection threshold at a significance level of $10^{-5}$. It is evident that as the number of reference stations increases, the separation between the fault-induced test statistics and the threshold becomes more pronounced.

**Figure 6:**
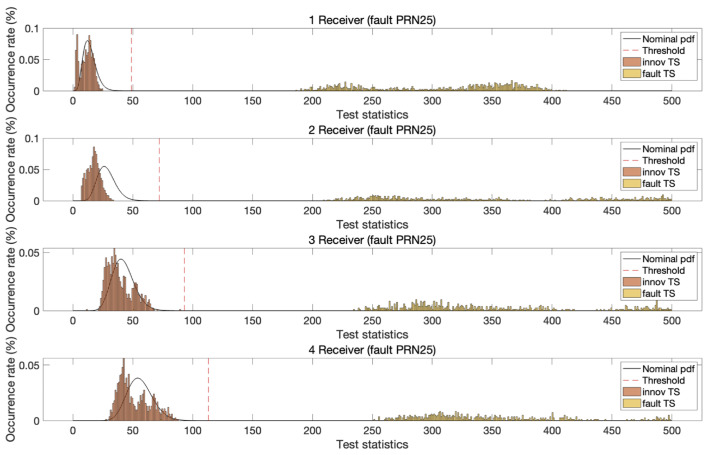
The chi-squared innovation test statistics (m^2^)of MR-RTK for PRN 25 GPS satellite under both nominal and fault conditions with a detection threshold at a significance level of $10^{-5}$. Although faults are deliberately injected at the same magnitude, the separation between the fault-induced test statistics and the threshold is more pronounced than that observed in Figure 5, due to differences in satellite geometry.

**Figure 7:**
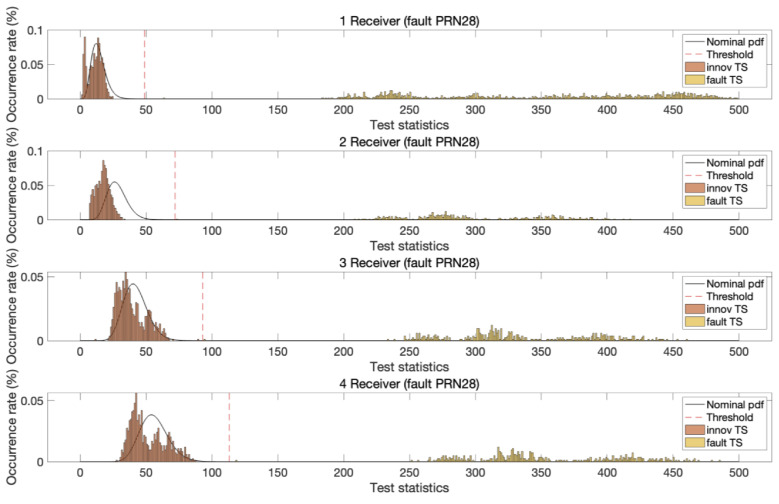
The chi-squared innovation test statistics (m^2^ of MR-RTK for PRN 28 GPS satellite under both nominal and fault conditions with a detection threshold at a significance level of $10^{-5}$. Although faults are deliberately injected at the same magnitude, the separation between the fault-induced test statistics and the threshold is more pronounced than that observed in Figure 5 and Figure 6, due to differences in satellite geometry.

To test the fault detection performance, pseudorange biases of 5 m and carrier phase biases of one cycle were introduced into the dual-frequency DD measurements. These step biases were applied as faults to both L1 and L2 signals, affecting GPS satellites with pseudorandom noise (PRN) values of 10, 25, and 28 in the test dataset. Faults were injected into one satellite at a time during each measurement epoch. Despite the identical bias magnitude applied to two satellites, the resulting test statistic distributions varied owing to differences in satellite geometry. Figure 5, Figure 6 and Figure 7 illustrate the test statistics $q_{reg}$ under nominal and fault conditions, along with the threshold for a false alert rate of $10^{-5}$ based on the central chi-squared distribution. These figures demonstrate that the experimentally obtained test statistics closely follow the theoretical chi-squared PDFs. In addition, as the number of reference stations increased, the chi-squared test statistic thresholds increased owing to the increased degrees of freedom in the test statistics. In all three cases, the test statistics under fault conditions exceeded the corresponding thresholds, and the separation between the threshold and test statistic increased with the number of reference stations.

**Figure 8:**
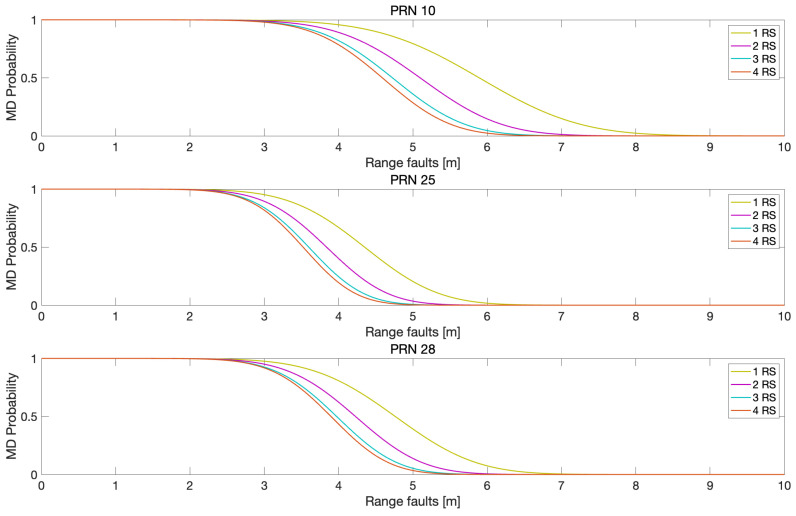
Assessment of missed detection (MD) probabilities in response to injected faults (m) for satellites with PRN of 10, 25, and 28. The horizontal axis represents the magnitude of faults injected into the pseudorange measurements for each satellite, while one-tenth of that fault magnitude was simultaneously applied to the corresponding carrier phase measurements. The results clearly demonstrate that the MD probabilities decrease as the number of reference stations increases across all three satellites.

**Figure 9:**
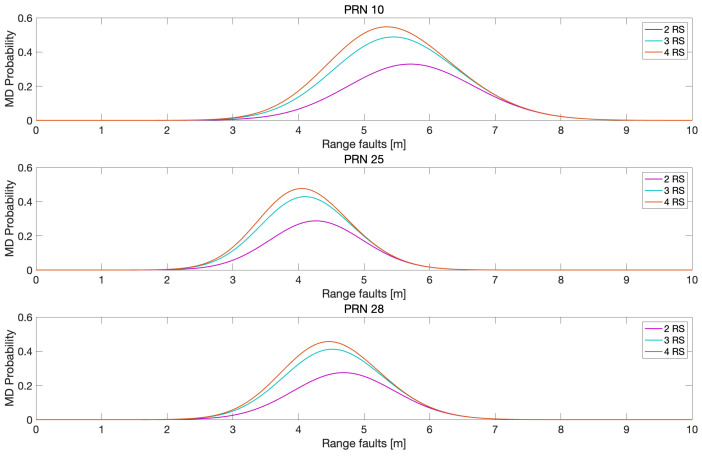
Reduction in missed detection (MD) probabilities relative to the single-reference-station case shown in Figure 8.The analysis reveals that the most significant reduction in MD probability—up to 0.54—occurs for a PRN of 10 when four reference stations are used.

To quantify the fault detection performance of MR-RTK, missed detection probabilities were computed for varying bias levels injected into the three satellites, as shown in Figure 8. In this analysis, range faults were introduced as step biases in pseudoranges, while one-tenth of the range fault magnitude was applied to carrier phase measurements at L1 and L2 frequencies. Similar to the trends observed in Figure 5, Figure 6 and Figure 7, Figure 8 shows that the missed detection probabilities decreased as the number of reference stations increased. The reduction in missed detection probabilities with the increase in reference stations compared with the case of using one reference station is illustrated in Figure 9. Table 3 lists the the maximum reduction of the missed detection probabilities in Figure 9.

## 5. Discussion

The results presented in the previous section clearly demonstrate that faults in user measurements can be detected more effectively as the number of reference stations increases. Indeed, GNSS augmentation networks—whether regional or nationwide—routinely utilize data from multiple reference stations for fault detection purposes [28]. In contrast, faults occurring on the user side are typically addressed using only the user’s own measurements or, in some cases, with the aid of auxiliary sensors. In carrier phase-based positioning, fault detection is particularly critical due to the high positioning accuracy requirements, as even small measurement faults can lead to incorrect integer ambiguity resolution. Such faults must, therefore, be identified with high confidence. The findings of this study suggest that the proposed MR-RTK architecture and associated monitoring technique offer a significant enhancement in detecting user-side measurement faults compared with conventional methods that rely solely on the measurements of a user and one reference station. The duration of rover maneuvering or raw measurement was intentionally kept short to better highlight differences in fault detection performance, as shorter measurement intervals minimize variations in satellite geometry.

## 6. Conclusions

In this study, we proposed an innovation-based fault-monitoring technique for an MR-RTK system. We demonstrated that GNSS measurements from multiple reference stations—with known baselines and resolved integer ambiguities—can be effectively used to detect faults in user measurements caused by excessive multipath or non-line-of-sight signals. The effectiveness of the proposed method was first validated through theoretical analysis, which showed increased separation between detection thresholds and noncentral parameter values in the chi-squared distribution. This theoretical insight was supported by experimental data collected using a dynamic rover and three publicly available reference stations. Faults were deliberately injected into the code and carrier phase measurements, and the results showed that increasing the number of reference stations led to greater separation between test statistics and detection thresholds, thereby reducing the likelihood of missed detection. Because faults can be more easily identified using the proposed method, we anticipate that subsequent fault identification and exclusion will be straightforward. In future research, we will focus on applying this MR-RTK fault detection and exclusion approach in urban road environments, where faults occur more frequently and are more difficult to detect. In addition, the real-time applicability of the proposed monitoring technique will be further explored, particularly in the context of cloud-based communication architectures.

## Figures and Tables

**Table 1:** Symbol and abbreviations used in the paper.

Symbol/Abbreviation	Description
$\phi_{i}$	DD carrier phase measurement vector for reference receiver *i*
$\rho_{i}$	DD pseudorange measurement vector for reference receiver *i*
$b_{i}$	Baseline vector between the user and reference receiver *i*
$n_{i}$	DD Integer ambiguity vector for reference receiver *i*
$b_{ij}$	Baseline vector between reference stations *i* and *j*
$n_{ij}$	DD integer ambiguities between reference stations *i* and *j*
Λ	Wavelength matrix
G	DD user-to-satellite line-of-sight geometry matrix for all frequencies
g	DD user-to-satellite line-of-sight geometry matrix for one frequency
⊗	Kronecker product operator
$e_{f}$	Column vector of 1s corresponding to the number of f
E(·)	Expectation operator (mean of a random variable)
*f*	Frequency
$h_{k}$	Nonlinear sensor model at epoch *k*
$y_{k}$	Measurement vector after applying a priori information at epoch *k*
$x_{k}$	EKF state vector at epoch *k*
$P_{k}$	State variance–covariance (VC) matrix at epoch *k*
$Q_{k-1}^{k}$	Process noise VC matrix from epoch k−1 to *k*
$F_{k-1}^{k}$	State transition matrix from epoch k−1 to *k*
$S_{k}$	Innovation VC matrix at epoch *k*
$H_{k}$	Measurement Jacobian matrix at epoch *k*
$R_{k}$	Measurement VC matrix at epoch *k*
$I_{m}$	Identity matrix with a dimension of m×m
$z_{k}$	Innovation vector at epoch *k*
$z_{k,n}$	Innovation vector at epoch *k* in nominal cases
$z_{k,f}$	Innovation vector at epoch *k* in fault cases
$f_{z}$	Innovation element occurred due to measurement fault
$q_{k}$	Innovation-based fault detection test statistic at epoch *k*
ν	Degree of freedom in a chi-squared distribution
τ	Noncentraility parameter of a chi-squared distribution
$\chi^{2}$	Chi-squared distribution

**Table 2:** Receiver model, baseline length from the master (HONG) and other reference stations, and GNSS signals available in the CORS network.

Station Name	Receiver Model	Baseline [km]	Used Signals
HONG	NovAtel OEM7700	-	GPS (L1/L2), GAL (E1)
YONS	TRIMBLE ALLOY 6.10	6.784	GPS (L1/L2), GAL (E1)
GANS	TRIMBLE ALLOY 6.10	7.810	GPS (L1/L2), GAL (E1)
GUMC	TRIMBLE ALLOY 6.10	10.504	GPS (L1/L2), GAL (E1)

**Table 3:** Impact of the number of reference stations (RSs) on the reduction in missed detection probability for selected PRN values.

Number of RS	PRN 10	PRN 25	PRN 28
2	0.32	0.29	0.27
3	0.49	0.43	0.41
4	0.55	0.47	0.46

## Data Availability

All data generated or analyzed during this study are included in this article. The raw data are available from the corresponding author upon reasonable request.
